# Controlling biomolecular condensates via chemical reactions

**DOI:** 10.1098/rsif.2021.0255

**Published:** 2021-06-30

**Authors:** Jan Kirschbaum, David Zwicker

**Affiliations:** Max Planck Institute for Dynamics and Self-Organization, Am Faßberg 17, 37077 Göttingen, Germany

**Keywords:** membraneless organelles, active droplets, reaction–diffusion systems

## Abstract

Biomolecular condensates are small droplets forming spontaneously in biological cells through phase separation. They play a role in many cellular processes, but it is unclear how cells control them. Cellular regulation often relies on post-translational modifications of proteins. For biomolecular condensates, such chemical modifications could alter the molecular interaction of key condensate components. Here, we test this idea using a theoretical model based on non-equilibrium thermodynamics. In particular, we describe the chemical reactions using transition-state theory, which accounts for the non-ideality of phase separation. We identify that fast control, as in cell signalling, is only possible when external energy input drives the reaction out of equilibrium. If this reaction differs inside and outside the droplet, it is even possible to control droplet sizes. Such an imbalance in the reaction could be created by enzymes localizing to the droplet. Since this situation is typical inside cells, we speculate that our proposed mechanism is used to stabilize multiple droplets with independently controlled size and count. Our model provides a novel and thermodynamically consistent framework for describing droplets subject to non-equilibrium chemical reactions.

## 1. Introduction

Biomolecular condensates are small droplets that structure the cell interior of eukaryotes [1,2] and prokaryotes [3–5]. They form by phase separation and participate in a wide range of cellular functions [6]: since they are chemically distinct from their surroundings, they can act as reaction centres [7,8], like the nucleolus inside the nucleus [9]. In particular, locally elevated concentrations can induce polymerization, as in microtubule branching [10] or in centrosomes [11,12], which additionally control the subcellular organization. Condensates can also store molecules to buffer fluctuations in gene expression [13] or to release them later when the condensate dissolves; examples of these include germ granules and the Balbiani body [14]. Condensates also help to detect changes in the environment externally, for example receptor clusters [15,16], and internally, for example stress granules [17]. In particular, transcriptional condensates actively regulate gene expression [18]. In all these examples, the cell controls the size, position or count of the biomolecular condensates [2,19].

The formation of biomolecular condensates can be described in the framework of liquid–liquid phase separation [20]. This implies that the droplet size is determined by the total amount of droplet material. Moreover, inevitable surface tension drives Ostwald ripening, which is a coarsening process dissolving smaller droplets in favour of larger ones, so that only a single droplet remains in thermodynamic equilibrium. The theory can also be used to predict how the droplet size depends on global parameters, such as temperature, pH and salt concentration [21–23]. Cells can directly control condensates by changing protein concentrations or molecular interactions [24,25]. The interactions are mainly dictated by the genetic sequence, which varies on evolutionary time scales. On cellular time scales, post-translational modifications can further adjust the interactions, enabling more dynamic regulation [26]. As an example, phosphorylating the carboxy-terminal domain (CTD) of RNA polymerase II dissolves CTD droplets *in vitro* [27]. More generally, chemical reactions, such as post-translational modifications, can affect the dynamics of droplets and explain how cells could regulate condensate size, location and count [28,29].

Theoretical studies of active droplets, which combine phase separation and chemical reactions, suggest that chemical reactions can suppress Ostwald ripening, leading to coexisting droplets of similar size [30,31] and even droplet division [32]. These studies described chemical reactions using fixed rate laws, which do not include the molecular interactions necessary for phase separation. Instead, a thermodynamically consistent theory is necessary to faithfully describe the interplay of phase separation with reactions. Earlier work in this direction [33–35] suggests that reactions need to be driven away from equilibrium to be effective.

Here, we present a minimal model of active droplets that combines non-equilibrium thermodynamics [36,37] and transition state theory [38,39] to describe the chemical reactions. It focuses on chemical potentials as key quantities and describes the non-equilibrium driving explicitly. We identify the conditions under which droplet size control is possible and determine the associated energetic cost. In the following, we build up the complete model by starting from passive liquid–liquid phase separation and then successively adding the reaction, the driving and enzymatic control.

## 2. Modelling phase separation with chemical transitions

We consider an incompressible, liquid mixture of a solvent and a chemical component that can exist in two different forms: a form *A*, which is soluble in the solvent, and an insoluble form *B*, which segregates from the solvent. The composition of the system is then given by the volume fractions *ϕ*_*i*_(***x***) of components *i* = *A*, *B* at each position ***x***. They evolve as

2.1a $$\partial_{t}\phi_{A}=-\nabla \cdot j_{A}-s$$

and

2.1b $$\partial_{t}\phi_{B}=-\nabla \cdot j_{B}+s,$$

where ***j***_*i*_ are diffusive fluxes and *s* is the reactive flux associated with the chemical transition A⇌B. We assume that the chemical component cannot leave the system, which implies that the normal fluxes ***n*** · ***j***_*A*_ and $n\cdot j_{B}$ vanish at the boundary with normal vector ***n***. Consequently, the total amount of the chemical component is conserved.

The diffusive and reactive fluxes, $j_{i}$ and *s*, can be described in the framework of non-equilibrium thermodynamics [36], which ensures that a closed system relaxes to thermodynamic equilibrium and that detailed balance is obeyed. One consequence is that the fluxes ***j***_*i*_ and *s* are related to the chemical potentials *μ*_*i*_(***x***) of the species *i* = *A*, *B*. In particular, the diffusive fluxes can be approximated by $j_{i}=-\sum_{j}\Lambda_{ij}\nabla \mu_{j}\;$, where the diffusive mobilities $\Lambda_{ij}$ form the symmetric, positive semi-definite Onsager matrix [36]. By contrast, such a linear approximation is inadequate for the reactive flux *s* [36] and we thus discuss a more detailed model below.

The chemical potentials *μ*_*i*_(***x***) describe how the free energy *F* of the system changes when a particle *i* = *A*, *B* replaces an equal volume of solvent at position ***x***. They are thus given by *μ*_*i*_ = *v*_*i*_ *δF*[*ϕ*_*A*_, *ϕ*_*B*_]/*δϕ*_*i*_, where we consider constant molecular volumes *v*_*i*_ and we assume *v*_*A*_ = *v*_*B*_. We focus on short-ranged molecular interactions, which typically drive phase separation in biological systems where electrostatic interactions are screened by counterions. In this case, the free energy of this isothermal system at temperature *T* can be expressed as

2.2 $$F[\phi_{A},\phi_{B}]=\int [f(\phi_{A},\phi_{B})-\sum_{i,j=A,B,C}\frac{\kappa_{ij}}{2}\nabla \phi_{i}\cdot \nabla \phi_{j}]dV,$$

where the integral is over the entire system of volume *V*_sys_. Here, *f* is the local free energy density, which governs phase separation [33], and *κ*_*ij*_ penalizes composition gradients, which results in surface tension effects [40]. As a concrete example, we consider the free energy density

2.3 $$\frac{\;f(\phi_{A},\phi_{B})}{k_{B}T}=\sum_{i}\frac{\phi_{i}}{v_{i}}\ln(\phi_{i})+\sum_{i}e_{i}\phi_{i}+\sum_{i,j}\frac{e_{ij}}{2}\phi_{i}\phi_{j},$$

where *k*_B_ is Boltzmann’s constant and *i*, *j* ∈ {*A*, *B*, *C*} using *ϕ*_*C*_ = 1 − *ϕ*_*A*_ − *ϕ*_*B*_. Here, the first term is the mixing entropy and the remaining terms capture enthalpic contributions [41,42]. In particular, *e*_*i*_ can be interpreted as internal energies, while *e*_*ij*_ = *e*_*ji*_ capture interactions. Since *A* molecules are soluble in the solvent, we assume for simplicity that they interact identically to the solvent (*e*_*AA*_ = *e*_*AC*_ = *e*_*CC*_ and *e*_*AB*_ = *e*_*BC*_). In the special case of a homogeneous system, the chemical potentials then read

2.4a $$\mu_{A}=k_{B}T\left(w_{A}+\ln\phi_{A}-\frac{v_{A}}{v_{C}}\ln\phi_{C}\right)$$

and

2.4b $$\mu_{B}=k_{B}T\left(w_{B}+\ln\phi_{B}-\frac{v_{B}}{v_{C}}\ln\phi_{C}-2\chi \phi_{B}\right),$$

where 2*χ* = *v*_*B*_(2*e*_*BC*_ − *e*_*BB*_ − *e*_*CC*_) is the Flory parameter capturing relevant interactions and *w_i_* = 1 − *v_i_*/*v_C_* + *v_i_*$\left(e_{i}-e_{C}+e_{iC}-e_{CC}\right)$ quantifies internal energies for *i* = *A*, *B*. In non-homogeneous systems, *μ*_*B*_ additionally contains the term $\kappa \nabla^{2}\phi_{B}$, which generates the surface tension. Here, *κ* = 2*k*_B_ *T*ℓ^2^*χ*, where ℓ is the interface width [40]. Equations (2.1) and (2.4) together form a typical model for describing phase separation without chemical reactions. We first briefly discuss this classical case and then proceed to examining how different models for the reaction fluxes *s* affect the droplet formation.

### 2.1. Amount of segregating material determines droplet size

**Figure 1.**
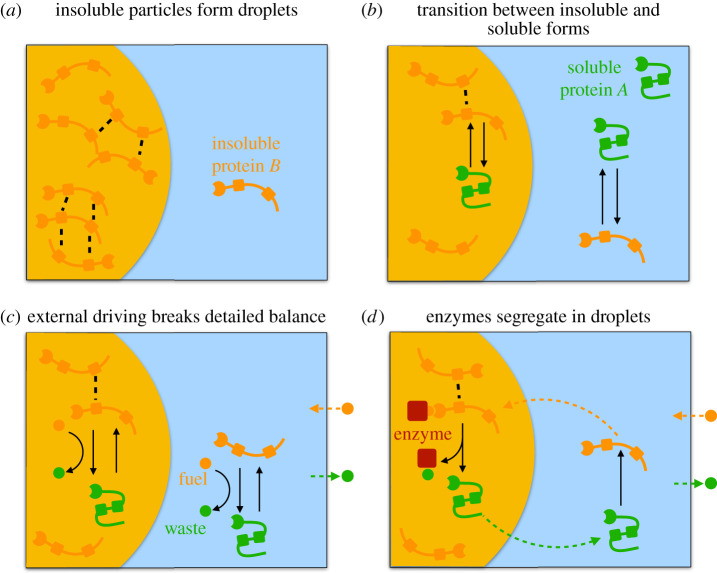
Schematic of the four discussed models: (*a*) *B* molecules (orange) with weak enthalpic interactions (black dashed lines) form droplets in the *C*-rich solvent phase (blue). (*b*) A spontaneous chemical transition (black arrow) between the segregating form *B* and the soluble form *A* (green) determines the amount of available droplet material *B*. (*c*) A second reaction (curved arrow) driven by the conversion of fuel *F* (orange circle) to waste *W* (green circle) can lead to a non-equilibrium stationary state when *F* and *W* are coupled to particle baths (arrows across box). (*d*) An enzyme segregating into droplets (red square) controlling the driven reaction causes cyclic diffusive fluxes (dashed arrows) in the system, which can stabilize multiple droplets in the same system.

**Figure 2.**
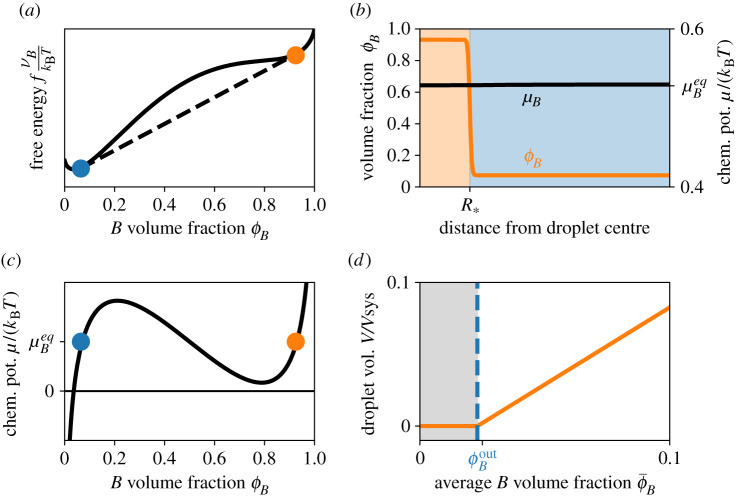
The amount of droplet material controls the size of passive droplets. (*a*) Free energy density *f* given by equation (2.3) as a function of the volume fraction *ϕ*_*B*_ (solid black line). A Maxwell construction (dashed black line) determines the fraction $\phi_{B}^{\mathrm{in}}$ inside droplets (orange circle) and the fraction $\phi_{B}^{\mathrm{out}}$ in the solvent (blue circle). (*b*) Equilibrium volume fraction *ϕ*_*B*_ (left axis) and associated chemical potential *μ*_*B*_ (right axis) as a function of the distance from the droplet centre. Shown is the numerical solution for a single droplet of radius $R_{*}$. (*c*) Chemical potential *μ*_*B*_ given by equation (2.4) as a function of the volume fraction *ϕ*_*B*_ of the droplet material. The chemical potentials are equal at the coexistence point (coloured dots). (*d*) Total volume *V* given by equation (2.5) of the droplet phase as a function of the average fraction ${\bar{\phi }}_{B}$ of the droplet material. Droplets do not form for ${\bar{\phi }}_{B}<\phi_{B}^{\mathrm{out}}$ (grey area). (*a*–*d*) Model parameters are *χ* = 3, *w*_*B*_ = 0.5, *v*_*B*_ = *v*_*C*_, ${\bar{\phi }}_{B}=0.08$ (in *b*) and *ϕ*_*A*_ = 0, so only *B* and *C* are present.

Without chemical reactions (*s* = 0; see figure 1*a*), droplets can form when the free energy *F* of the demixed system is lower than that of the homogeneous system. This is the case if *χ* is large enough (figure 2*a*) while the internal energies *w*_*i*_ are irrelevant since the total amount of each species is conserved [33]. In equilibrium, the diffusive flux ***j***_*B*_ vanishes and the chemical potential *μ*_*B*_ is homogeneous, while *ϕ*_*B*_ can vary strongly (figure 2*b*,*c*). The respective equilibrium fractions $\phi_{B}^{\mathrm{in}}$ and $\phi_{B}^{\mathrm{out}}$ inside and outside of the droplet are given by a tangent construction (figure 2*a*) [40]. They are constant and do not depend on the total composition of the system if the fraction of *A* is small (*ϕ*_*A*_ ≪ 1). Without reactions, there are only two equilibrium states: either everything is mixed or a single droplet enriched in *B* forms. Even if multiple droplets form initially, for example because of nucleation, surface tension effects drive coarsening by Ostwald ripening [43] or coalescence, so that all droplets merge into one [33]. The volume *V* of the droplet follows from material conservation and reads

2.5 $$V=\frac{{\bar{\phi }}_{B}-\phi_{B}^{\mathrm{out}}}{\phi_{B}^{\mathrm{in}}-\phi_{B}^{\mathrm{out}}}V_{\mathrm{sys}},$$

where ${\bar{\phi }}_{B}=V_{\mathrm{sys}}^{-1}\int \phi_{B}\;dV$ is the average fraction of *B* in the system. Note that the droplet can only exist when ${\bar{\phi }}_{B}>\phi_{B}^{\mathrm{out}}$. All excess material beyond $\phi_{B}^{\mathrm{out}}$ concentrates in the droplet, so *V* grows linearly with ${\bar{\phi }}_{B}$ (figure 2*d*). A biological cell can thus regulate whether a droplet exists and how large it gets by controlling the total amount of *B*. Protein amounts can be changed by production and degradation, although this is a costly and slow process. Moreover, *V* depends on the interaction parameter *χ*, which is a function of, for example, temperature, pressure, pH and solvent composition. These parameters are either external to the cell or affect many other processes, so they are not ideal to regulate a specific droplet. Taken together, this analysis shows that additional processes are necessary to control phase separation effectively.

### 2.2. Chemical reactions control amount of segregating material

A chemical transition that modifies the physical properties of the droplet material can affect droplet formation. Our model captures this when we allow transitions between the soluble form *A* and the segregating form *B* of the material. The associated reaction rate *s* is given by the difference in the rate $s^{f}$ of the forward reaction *A* → *B* and the rate $s^{b}$ of the opposite direction, $s=s^{f}-s^{b}$. In the simplest case, the transition A⇌B does not require external energy input (figure 1*b*), implying the detailed balance condition [33]

2.6 $$\frac{s^{f}}{s^{b}}=\exp\left(\frac{\mu_{A}-\mu_{B}}{k_{B}T}\right).$$

Chemical equilibrium (*s* = 0) is thus reached when *μ*_*A*_ = *μ*_*B*_.

In the simple case of a homogeneous system, the equilibrium state can be characterized by the fractions $\phi_{A}^{\mathrm{eq}}$ and $\phi_{B}^{\mathrm{eq}}$ of the two forms. However, since the total fraction *ϕ*_+_ = *ϕ*_*A*_ + *ϕ*_*B*_ of the component is conserved, it is convenient to also discuss the equilibrium constant $K=\phi_{B}^{\mathrm{eq}}/\phi_{A}^{\mathrm{eq}}$. Using the chemical equilibrium (*μ*_*A*_ = *μ*_*B*_) and equation (2.4), we find

**Figure 3.**
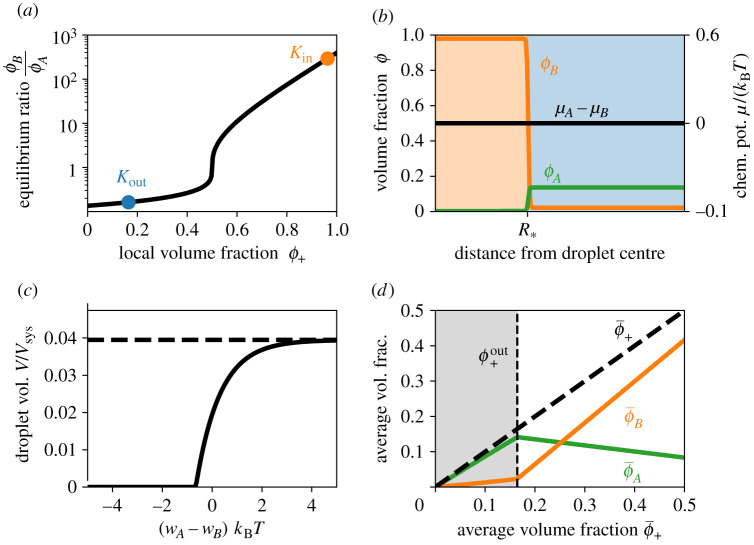
Chemical equilibrium sets the amount of droplet material. (*a*) The equilibrium ratio *K* of the fractions of soluble and phase-separating forms given by equation (2.7) as a function of the total protein fraction *ϕ*_+_ = *ϕ*_*A*_ + *ϕ*_*B*_. The respective values *K*_in_ and *K*_out_ inside and outside the droplet are indicated. (*b*) Numerically determined volume fractions *ϕ*_*A*_ and *ϕ*_*B*_ (left axis) and the associated chemical potential difference *μ*_*A*_ − *μ*_*B*_ (right axis) as a function of the distance from the centre of a droplet of radius $R_{*}$. The composition of the droplet (orange shaded area) can differ strongly from that of the solvent (blue shaded area) even at equilibrium (*μ*_*A*_ = *μ*_*B*_). (*c*) Total fraction *V*/*V*_sys_ occupied by droplets given by equation (2.8) as a function of the difference *w*_*A*_ − *w*_*B*_ between the internal energy of *A* and *B*. The dashed line marks the maximal volume, where all proteins are in form *B*. (*d*) Average volume fractions of *A* (green line) and *B* (orange line) resulting from *K* given in equation (2.7) as a function of the total average protein volume fraction ${\bar{\phi }}_{+}$ in the system. Without droplets (grey area), almost all protein is in the soluble *A* form, while the opposite is true for large droplets. (*a*–*d*) Model parameters are *χ* = 4, *w*_*A*_ − *w*_*B*_ = 2, *v*_*A*_ = *v*_*B*_ = *v*_*C*_ (in *a*–*c*), ${\bar{\phi }}_{+}=0.2$ (in *b*) and ${\bar{\phi }}_{+}=0.06$ (in *c*).

2.7 $$K=\exp(w_{A}-w_{B}+2\chi \phi_{B}),$$

which shows that *K* is strongly affected by the difference *w*_*A*_ − *w*_*B*_ of the internal energies of *A* and *B*. Note that *K* is only a constant for an ideal solution (*χ* = 0). For a non-ideal system, *K* depends on the total fraction *ϕ*_+_, such that *K* is larger when there is more material (figure 3*a*). Taken together, this analysis shows that the chemical equilibrium depends on the environment.

The only inhomogeneous equilibrium state of the system is again a single droplet enriched in *B*. The analysis of the inhomogeneous state implies that the ratio $\phi_{B}^{\mathrm{eq}}/\phi_{A}^{\mathrm{eq}}$ is larger inside the droplet than outside. This is because the droplet environment favours *B* over *A*. Note that *A* is enriched outside the droplet for the chemical potentials given by equation (2.4) (figure 3*b*), but more general choices of *e*_*ij*_ can enrich *A* inside the droplet. For our system, *ϕ*_*A*_ is thus dilute in both phases in the common case that the system mostly consists of solvent (${\bar{\phi }}_{+}\ll 1$), where ${\bar{\phi }}_{+}=V_{\mathrm{sys}}^{-1}\int \phi_{+}dV$ denotes the conserved total fraction of *A* and *B*. In this case, we can determine the equilibrium fractions of *B* from the tangent construction given in figure 2*a* since *ϕ*_*A*_ ≪ 1 everywhere. We can then use $K_{\mathrm{in}}=K(\phi_{B}^{\mathrm{in}})$ and $K_{\mathrm{out}}=K(\phi_{B}^{\mathrm{out}})$ to determine the total fraction *ϕ*_+_ inside and outside the droplet, $\phi_{+}^{\mathrm{in}}=(1+K_{\mathrm{in}}^{-1})\phi_{B}^{\mathrm{in}}$ and $\phi_{+}^{\mathrm{out}}=(1+K_{\mathrm{out}}^{-1})\phi_{B}^{\mathrm{out}}$. The conservation of $\;{\bar{\;\phi }}_{+}$ then implies that the droplet volume *V* is given by

2.8 $$V=\frac{{\bar{\phi }}_{+}-\phi_{+}^{\mathrm{out}}}{\phi_{+}^{\mathrm{in}}-\phi_{+}^{\mathrm{out}}}V_{\mathrm{sys}}.$$

Similarly to the case without chemical reactions, a droplet can only form when ${\bar{\phi }}_{+}>\phi_{+}^{\mathrm{out}}$ and the total amount exceeding the threshold determines the droplet volume. However, the internal energy difference *w*_*A*_ − *w*_*B*_ now also affects the droplet volume (figure 3*c*). This is mainly because it changes the equilibrium constant *K*_out_ and thus $\phi_{+}^{\mathrm{out}}$. Consequently, external parameters, such as temperature and pH, can now also affect droplet formation via the internal energies, thus allowing for a potentially stronger response.

The chemical reactions clearly influence the droplet formation and thus the overall composition in the system. In particular, the relative amounts of *A* and *B* strongly depend on whether droplets form or not. Figure 3*d* shows that the amount of *B* in the system increases significantly when the total fraction ${\bar{\phi }}_{+}$ exceeds the threshold $\phi_{+}^{\mathrm{out}}$ so droplets form.

We showed that the chemical transition allows for more detailed control of droplet formation. However, similar to the case without chemical reactions, changing the total amount is costly and slow while the involved energy difference *w*_*A*_ − *w*_*B*_ mainly depends on external parameters. This is because the equilibrium states in both cases are governed by the free energy. In particular, kinetic parameters are irrelevant and droplets cannot be controlled enzymatically.

### 2.3. Driven reactions allow enzymatic control of droplets

We next extend our system by allowing the transition A⇌B to also be driven by an external energy input (figure 1*c*). In particular, we introduce a second reaction, A+W⇌B+F, where *F* and *W*, respectively, denote fuel and waste molecules. A typical biological example is ATP and ADP, where the hydrolysis of ATP liberates about 15–30 *k*_B_*T* [44]. For simplicity, we consider the case where *F* and *W* are dilute and homogeneously distributed, so they do not affect phase separation directly. To keep the system away from equilibrium, we assume that the chemical potential difference Δ*μ* = *μ*_*F*_ − *μ*_*W*_ > 0 is constant, e.g. because of ATP regeneration. Taken together, Δ*μ* can be interpreted as a ubiquitous external energy source.

The driven reaction obeys the detailed balance condition [33]

2.9 $$\frac{s_{2}^{f}}{s_{2}^{b}}=\exp\left(\frac{\mu_{A}-\mu_{B}-\Delta \mu }{k_{B}T}\right),$$

where $s_{2}^{f}$ and $s_{2}^{b}$ are the forward and backward rates. The net rate $s_{2}=s_{2}^{f}-s_{2}^{b}$ of the driven reaction thus vanishes when *μ*_*A*_ − *μ*_*B*_ = Δ*μ*. This condition is incompatible with the chemical equilibrium of the passive reaction, *μ*_*A*_ = *μ*_*B*_, discussed in the previous section. This implies that the driven system cannot reach thermodynamic equilibrium.

To understand the behaviour of the driven system, we first consider stationary states where the total reactive flux, *s* = *s*_1_ + *s*_2_, vanishes. Here, $s_{1}=s_{1}^{f}-s_{1}^{b}$ is the rate associated with the passive reaction, where $s_{1}^{f}$ and $s_{1}^{b}$ obey the detailed balanced condition given by equation (2.6). Taken together, the condition *s* = 0 requires that the stationary state chemical potentials $\mu_{A}^{*}$ and $\mu_{B}^{*}$ obey

**Figure 4.**
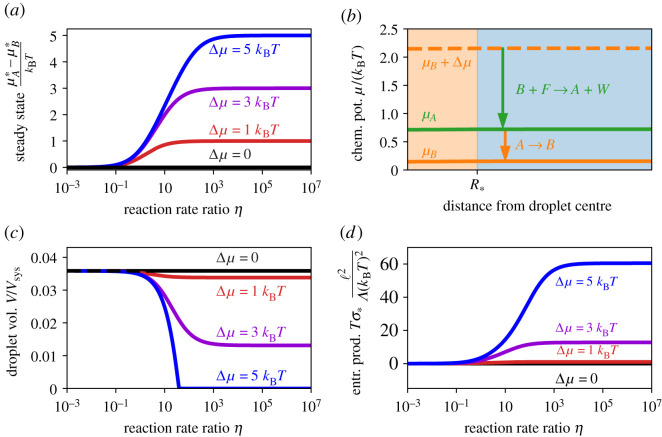
A driven transition affects the chemical equilibrium. (*a*) Chemical potential difference $\mu_{A}^{*}-\mu_{B}^{*}$ between *A* and *B* given by equation (2.10) as a function of the ratio $\eta =\alpha_{1}^{-1}\alpha_{2}\exp(\mu_{F}/k_{B}T)$ of the reaction rate of the passive and driven reactions in the stationary state for various driving strengths Δ*μ*. (*b*) Numerically determined chemical potentials of *A* (green line) and *B* (solid orange line) as a function of the radial distance from the centre of a droplet of radius $R_{*}$. The dashed orange line marks the chemical potential difference of the driven reaction, indicating that it spontaneously produces *A* (green arrow), which is then turned back into *B* by the passive reaction (orange arrow). Although the system is driven out of equilibrium, the reactions balance locally, so $\nabla \mu_{i}=0$. (*c*) Total fraction *V*/*V*_sys_ occupied by droplets as a function of *η* for different driving strengths Δ*μ*. *V* is calculated from equations (2.7), (2.8) and (2.10) as described in the main text. Droplets only vanish completely if the driving is strong enough. (*d*) Entropy production rate $T\sigma_{*}=-V_{\mathrm{sys}}s_{2}\Delta \mu$ in the stationary state as a function of *η* for different driving strengths Δ*μ*. The analytical results were obtained using equation (2.9) as described in the main text. (*a*–*d*) Model parameters are *χ* = 4, *w*_*A*_ − *w*_*B*_ = 3, *v*_*A*_ = *v*_*B*_ = *v*_*C*_ and *ϕ*_+_ = 0.06; (*b* only) Δ*μ* = 2 *k*_B_*T*, *η* = 1, *w*_*A*_ − *w*_*B*_ = 4.

2.10 $$\mu_{A}^{*}-\mu_{B}^{*}=\Delta \mu -k_{B}T\ln\left[\frac{\exp(\Delta \mu /k_{B}T)+\eta }{1+\eta }\right],$$

where $\eta =s_{2}^{b}/s_{1}^{b}$ is the ratio of the backward reaction rates. Note that this condition corresponds to the passive case ($\mu_{A}^{*}=\mu_{B}^{*}$) for *η* = 0, while the driven reaction dominates ($\mu_{A}^{*}-\mu_{B}^{*}=\Delta \mu$) for *η* → ∞ (figure 4*a*). In general, we have $0\leq \mu_{A}^{*}-\mu_{B}^{*}\leq \Delta \mu$, so that the passive reaction creates the segregating form *B* while the driven reaction destroys it.

In general, the backward rates $s_{i}^{b}$ depend on composition, since they describe the kinetics of the chemical reactions [34]. A simple model for chemical reactions is transition state theory [38,39], where the forward and backward rates only depend on the chemical potentials of the reactants and products, respectively. Using this theory, we find $s_{1}^{b}=\alpha_{1}\exp(\mu_{B}/k_{B}T)$ and $s_{2}^{b}=\alpha_{2}\exp[(\mu_{B}+\mu_{F})/k_{B}T]$, where *α*_*i*_ are constant pre-factors that can be influenced by enzymes; see the electronic supplementary material. This implies that $\eta =\alpha_{2}\alpha_{1}^{-1}\exp(\mu_{F}/k_{B}T)$, and thus also $\mu_{A}^{*}-\mu_{B}^{*}$, are constant (see equation (2.10)). Taken together with equation (2.1), we thus find that all stationary states with *s* = 0 must have homogeneous chemical potentials (figure 4*b*).

The driven system can be mapped to the system with passive reactions by altering the internal energies, $w_{B}\mapsto w_{B}+(\mu_{A}^{*}-\mu_{B}^{*})/k_{B}T$. Consequently, this system possesses the same stationary states as the passive system, so that at most a single droplet can form and its volume is given by equation (2.8). However, the driven chemical reaction can now be used to control the droplet volume, e.g. by enzymatic activity. For instance, increased activity of an enzyme that catalyses the driven reaction corresponds to an increase in *α*_2_. This results in an increase in *η*, $\mu_{A}^{*}-\mu_{B}^{*}$, *s*, *K*^−1^ and $\phi_{+}^{\mathrm{out}}$, which leads to a smaller droplet volume *V*; see figure 4*c*. Equivalently, raising the external potential Δ*μ* also reduces *V*. In particular, any change that increases $\phi_{+}^{\mathrm{out}}$ beyond the average fraction ${\bar{\phi }}_{+}$ of available material will dissolve all droplets. Note that this dissolution by enzymatic reactions happens without degrading the material, so droplets could re-form quickly when the original conditions are restored. However, the potential for this quick response comes at the energetic cost, quantified by the entropy production (figure 4*d*), of keeping the droplets dissolved [31].

We showed that at most a single droplet can be stable when the net flux of the chemical reactions vanishes everywhere (*s* = 0). To also regulate the droplet count, we thus need inhomogeneous states where *s* ≠ 0. However, we show in the electronic supplementary material that there are no stationary states with *s* ≠ 0 if *η* and Δ*μ* are the same everywhere. Consequently, *η* or Δ*μ* must vary in space to have multiple stable droplets. This could be achieved by imposing spatial heterogeneity, e.g. by producing the fuel or enriching enzymes at particular locations, which would be reflected in the droplet arrangement. Alternatively, the fuel or enzymes can segregate into the droplets spontaneously, which is observed experimentally [27].

### 2.4. Segregated enzymes can control droplet size and count

The main idea to control droplets is to use an enzyme that regulates the chemical transition and segregates into droplets. As an example, we consider an enzyme *E* that affects the driven reaction (figure 1*d*). In the simplest case, the rate *s*_2_ of this reaction is proportional to the volume fraction *ϕ*_*E*_ of the enzyme,

2.11 $$s_{2}=\alpha_{2}^{E}\phi_{E}\left[\exp\left(\frac{\mu_{A}+\mu_{W}}{k_{B}T}\right)-\exp\left(\frac{\mu_{B}+\mu_{F}}{k_{B}T}\right)\right],$$

which follows from equation (2.9) and transition state theory (see the electronic supplementary material). Here, $\alpha_{2}^{E}$ is a constant pre-factor, so that this case is equivalent to the one discussed in the previous section if *ϕ*_*E*_ is homogeneous.

The distribution of the enzyme will be inhomogeneous if it segregates into droplets. We model this by introducing an additional Flory parameter *χ*_*E*_, which describes the interaction of the enzyme with the other components (see the electronic supplementary material). For simplicity, we consider dilute enzyme concentrations, so the coexisting concentrations of the droplet material *B* at the interface are not significantly affected. Consequently, *χ*_*E*_ controls how strongly the enzyme segregates into the droplet [45],

2.12 $$\frac{\phi_{E}^{\mathrm{in}}}{\phi_{E}^{\mathrm{out}}}\approx e^{\chi_{E}(\phi_{B}^{\mathrm{in}}-\phi_{B}^{\mathrm{out}})}$$

(see the electronic supplementary material). In particular, the enzyme is homogeneously distributed for *χ*_*E*_ = 0, corresponding to the case discussed in the previous section.

**Figure 5.**
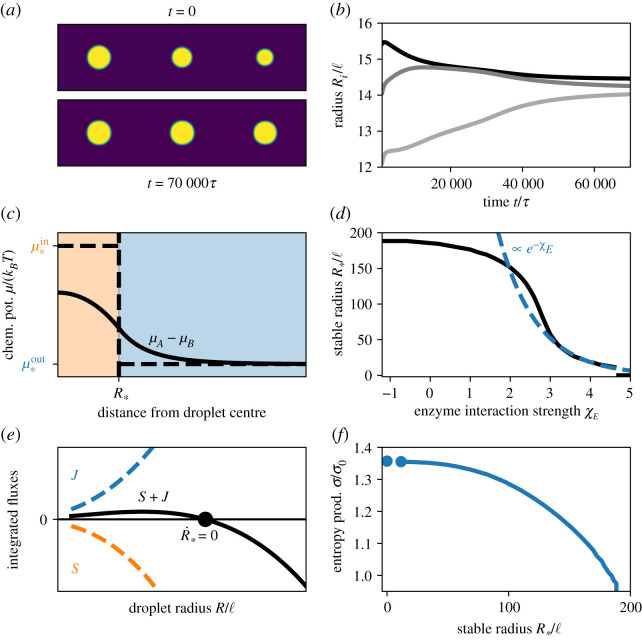
A segregated enzyme can control and stabilize multiple droplets. (*a*) Snapshots of a numerical simulation of three droplets in a cylindrical geometry at two time points. Simulation parameters are *χ* = *χ*_*E*_ = 4, *w*_*A*_ − *w*_*B*_ = 2, *v*_*A*_ = *v*_*B*_ = *v*_*C*_, Δ*μ* = 10*k*_B_*T*, $\Lambda_{ij}=\delta_{ij}\;k_{B}T\ell^{2}/\tau$, *α*_1_ = 10^−3^ *τ*^−1^, *η* = 3 and ${\bar{\phi }}_{+}=0.25$. (*b*) Droplet radii *R*_*i*_ of the simulation in (*a*) as a function of time. (*c*) Numerical solutions of the steady-state chemical potential difference between *A* and *B* as a function of the distance from the droplet centre. The chemical potential gradient does not vanish across the interface between the droplet (orange) and the solvent phase (blue), implying diffusive fluxes. Far away from the droplet, the reactions cancel each other (*s* = 0), while *s* < 0 in the droplet and *s* > 0 in the solvent close to the interface. (*d*) Stable droplet radius $R_{*}$ as a function of the interaction parameter *χ*_*E*_ for the enzyme. The numerical result (black solid line) is compared with the scaling (blue dashed line) for strong segregation discussed in the main text. (*e*) Numerically determined reaction fluxes integrated over the droplet volume (*S*, orange dashed line), the solvent volume (*J*, blue dashed line) and the entire system (black solid line) as a function of the droplet radius. The fluxes *S* and *J* are equal and opposite at the stationary state (black dot). (*f*) Entropy production rate $T\sigma =-\int s_{2}\Delta \mu \;dV$ as a function of $R_{*}$ for the situation shown in (*d*). The data are normalized to the entropy production *σ*_0_ obtained for *χ*_*E*_ = 0, where the enzyme distributes homogeneously. (*c*–*f*) Results are obtained numerically using *χ* = 4, ${\bar{\phi }}_{+}=0.06$, ${\bar{\phi }}_{E}=0.001$, $\Lambda_{ij}=\delta_{ij}\;k_{B}T\ell^{2}/\tau$, *α*_1_ = 5 × 10^−4^ *τ*^−1^, *η* = 0.2, *w*_*A*_ − *w*_*B*_ = 4, *v*_*A*_ = *v*_*B*_ = *v*_*C*_ and Δ*μ* = 5 *k*_B_*T*. Further details are given in the electronic supplementary material.

The enzyme is enriched in the droplet when *χ*_*E*_ > 0. In this case, the driven reaction can stabilize multiple droplets at the same size; see figure 5*a*–*b*. To understand this behaviour, we analyse a single droplet in a large system. Figure 5*c* shows that the chemical potentials of *A* and *B* are now inhomogeneous even in the stationary state. This implies diffusive fluxes, which are driven by the non-equilibrium chemical reactions: effectively, inside the droplet, the driven chemical reaction turns the segregating form *B* into the soluble form *A*, while form *A* transitions back to *B* spontaneously outside. The resulting imbalances between the inside and the outside are compensated by the diffusive fluxes. Consequently, the chemical reactions drive a cycle of diffusive fluxes (figure 1*d*).

The numerical simulations also show that the stable droplet radius $R_{*}$ decreases with larger enzyme segregation (larger *χ*_*E*_) (figure 5*d*). In the stationary state, the diffusive influx *J* of *B* towards the droplet is balanced by the reactive flux *S* of *B* → *A* inside the droplet (*J* = *S*). In the simplest case, *J* is diffusion limited, *J* ≈ *a*_1_*R*, while the reaction is homogeneous in the droplet, implying *S* ≈ *a*_3_exp(*χ*_*E*_)*R*^3^ (see the electronic supplementary material). Consequently, the stable radius scales as $R_{*}∼\exp(-\frac{1}{2}\chi_{E})$. In more realistic cases, the reaction affects the influx *J*, leading to *J* ≈ *a*_2_*R*^2^, which implies $R_{*}∼\exp(-\chi_{E})$ (figure 5*e*). In both cases, a stable droplet size exists when the fluxes *J* and *S* are equal (figure 5*e*).

The droplet size regulation depends on the non-equilibrium chemical reactions, which maintain a chemical potential difference between the droplet and its surrounding (figure 5). We observe that the associated entropy production rate *σ* increases for smaller radii $R_{*}$ (figure 5*f*). This suggests that keeping droplets small consumes more fuel *F*. In particular, preventing droplet formation ($R_{*}=0$) is costly. Conversely, larger droplets require smaller entropy production, although it is still non-zero, similar to the case in the previous section. The fact that droplets reach a stable size implies that multiple droplets can coexist in a larger system. Since all droplets attain the same volume $V_{*}$, the number *N* of droplets is simply $N=V/V_{*}$, where the total volume *V* of the droplet phase can be approximated by equation (2.8). In particular, *V* depends on the total fraction ${\bar{\phi }}_{+}$ of *A* and *B*, the Flory parameter *χ*, the internal energies *w*_*A*_ − *w*_*B*_, the driving strength Δ*μ* and the reaction rate ratio *η*. Conversely, the stable radius $V_{*}$ is additionally controlled by *χ*_*E*_, so the droplet count *N* and the individual volume $V_{*}$ can be adjusted independently.

## 3. Discussion

We introduced a model that explains how chemical reactions can control liquid-like droplets. In particular, we identified three ingredients necessary for effective size control: (i) the chemical modification of the droplet material must convert it to a soluble form, (ii) this modification must involve a driven reaction using a chemical fuel, and (iii) the reaction dynamics must differ inside and outside the droplet, e.g. by localizing enzymes appropriately. The fuel, combined with the imbalance of the reaction, maintains a chemical potential difference between the inside and the outside, which results in sustained diffusive fluxes. This effectively removes droplet material from the droplet while producing it outside, which explains the stable size of this externally maintained droplet [33]. In an alternative interpretation, the enzymes enriching in the droplet inhibit further growth, which we already identified as a common motif for size control in biological cells [29]. The stable droplet size $R_{*}$ predicted by our model is mainly governed by the chemical transition inside the droplet (see the electronic supplementary material). In particular, $R_{*}∼{\left[3D\Delta c/(k_{0}c_{E}^{\mathrm{in}})\right]}^{1/2}$ where *D* ≈ 1 μm^2^ s^−1^ is a typical diffusivity [46], while the other parameters can vary widely [44]. Here, Δ*c* quantifies the concentration variation of droplet material *B* in the dilute phase, *k*_0_ is the catalytic rate constant and $c_{E}^{\mathrm{in}}$ is the enzyme concentration inside the droplet. For strong reactions (*k*_0_ ≈ 100 s^−1^) and strong enzyme segregation ($\Delta c/c_{E}^{\mathrm{in}}\approx 0.1$), we find very small droplets ($R_{*}\approx 0.05\;\mu m$). Conversely, droplets are much larger ($R_{*}\approx 17.3\;\mu m$) for weaker reactions (*k*_0_ ≈ 0.1 s^−1^) and moderate segregation ($\Delta c/c_{E}^{\mathrm{in}}=10$). Consequently, droplets can be stabilized on all length scales relevant to biological cells. In particular, $R_{*}$ is governed by intrinsic model parameters and is thus independent of system size, similar to other theoretical predictions from combining phase separation with chemical reactions [30,31,34,47]. Whether droplets form and how large they get mainly depends on the available amount of droplet material. Our model reveals that this key quantity can be regulated on many scales in biological cells: adapting the genetic sequence on evolutionary time scales affects the internal energies of the soluble and segregating forms, thus influencing the fraction of droplet material (figure 3*d*). On the time scale of minutes to hours, protein production and degradation affect the overall composition (figure 2*d*). Faster time scales are accessible using active processes: by activating and deactivating enzymes, the cell can regulate the reaction rates *α*_1_ and *α*_2_ and thus the balance between the two forms. Moreover, our analysis shown in figure 4*d* indicates that the stable droplet size is very sensitive to the ratio *η* = *α*_2_/*α*_1_, implying that even small changes in these rates can have a significant impact. This active regulation allows cells to quickly adapt their biomolecular condensate in response to internal and external signals [15–17]. Moreover, the continuous turnover of droplet material could prevent the observed ageing of biomolecular condensates [48,49].

Our model unveils the required ingredients for droplet size regulation since it obeys thermodynamic constraints, in contrast to our earlier theory [30]. Similar to electro-chemical systems [35], the chemical reactions in our system cannot be described by the law of mass action since phase-separating solutions are non-ideal. In particular, the associated equilibrium constants differ inside and outside the droplet (figure 3*a*). To investigate this further, our theory could be extended to client chemical reactions [45], multi-component droplets [24], complex multi-layered droplets [40,50,51] and multiple different droplets affecting each other [52], which are all relevant in biological cells. Moreover, it is unclear how active droplets interact with other subcellular structures, such as the cytoskeleton [53], or generally with the elastic properties of their surrounding [54,55]. It will be interesting to test our ideas with engineered condensates [56] using fuelled chemical reactions [57].

## Data accessibility

Code needed to reproduce the results is available at Zenodo [58]. All other data are available in the manuscript or the electronic supplementary material.
